# Tipping Points in Seaweed Genetic Engineering: Scaling Up Opportunities in the Next Decade

**DOI:** 10.3390/md12053025

**Published:** 2014-05-22

**Authors:** Hanzhi Lin, Song Qin

**Affiliations:** 1 Environmental Biophysics and Molecular Ecology Program, Institute of Marine and Coastal Sciences, Rutgers University, 71 Dudley Road, New Brunswick, NJ 08901, USA; 2 Key Lab of Coastal Biology and Bio-resource Utilization, Yantai Institute of Coastal Zone Research, Chinese Academy of Sciences, 17 Chunhui Road, Yantai 264003, China

**Keywords:** seaweed, macroalgae, genetic engineering, GMO, gene flow, bio-safety, marine algae, transgenic algae, expression system

## Abstract

Seaweed genetic engineering is a transgenic expression system with unique features compared with those of heterotrophic prokaryotes and higher plants. This study discusses several newly sequenced seaweed nuclear genomes and the necessity that research on vector design should consider endogenous promoters, codon optimization, and gene copy number. Seaweed viruses and artificial transposons can be applied as transformation methods after acquiring a comprehensive understanding of the mechanism of viral infections in seaweeds and transposon patterns in seaweed genomes. After cultivating transgenic algal cells and tissues in a photobioreactor, a biosafety assessment of genetically modified (GM) seaweeds must be conducted before open-sea application. We propose a set of programs for the evaluation of gene flow from GM seaweeds to local/geographical environments. The effective implementation of such programs requires fundamentally systematic and interdisciplinary studies on algal physiology and genetics, marine hydrology, reproductive biology, and ecology.

## 1. Introduction

Seaweeds (marine macroalgae) are plant-like organisms encompassing macroscopic, multicellular, benthic marine algae. The term comprises red, brown and green algae, which are classified according to the thallus color derived from their dominant pigments (phycoerythrin and phycocyanin in red algae, *Chlorophyll* a and b in green algae, and fucoxanthin in brown algae). They generally live attached to hard substrates (such as rocks) in coastal areas, although some brown algae in Laminariales and red algae in Corallinales can live at depths of several or occasionally nearly a hundred meters below the sea surface [1]. A number of species/populations have adapted to be free-floating (*Sargassum* and *Ulva*) via changes of their intercellular gas sacs to maintain their favored depth in the water. There are approximately 10,000 species of seaweed throughout the world; however, seaweeds are generally regarded as a polyphyletic group that does not have a genetically common multicellular ancestor. They originated through multiple endosymbiotic events during the course of geological time. Generally speaking, green and red algae originated from a primary endosymbiosis when a eukaryotic host cell acquired an ancestral cyanobacterium as its plastid to form a primary symbiotic oxygenic eukaryote [2]. Brown algae derived from a secondary endosymbiosis, whose ancestor historically possessed a cryptic green algal endosymbiont that was subsequently replaced by a red algal chloroplast [3]. Due to their genetically polyphyletic origin, they are now classified in different kingdoms (brown algae are in the Kingdom Chromista, green algae and red algae are in the Kingdom Plantae) [4,5], although all are referred to as seaweed in assemblage.

**Figure 1:**
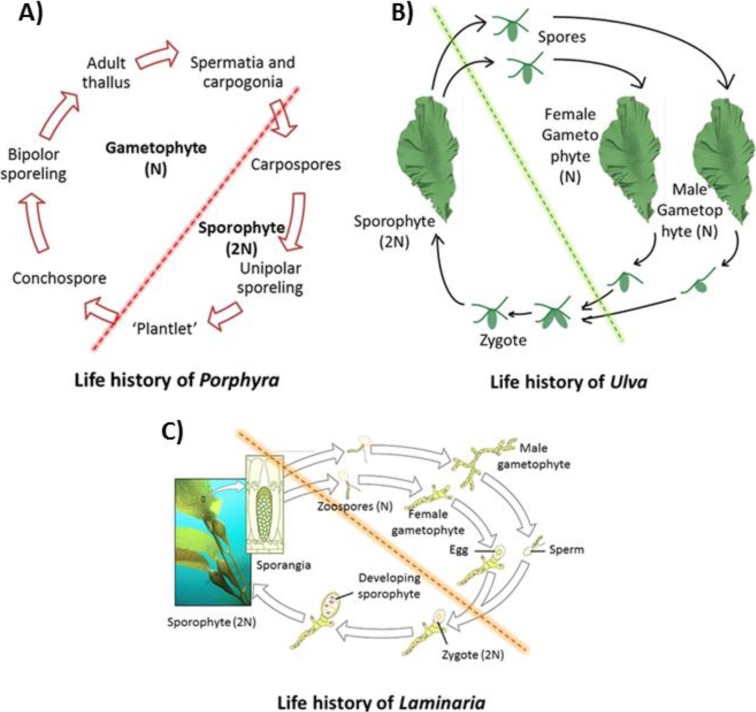
Life histories of main three groups of seaweeds. (**A**) Life history of *Porphyra*, redrew and based on [6]. (**B**) Life history of *Ulva*, redrew and based on [15]. (**C**) Life history of *Laminara*, adapted from [16], with permission from openstax of Rice University.

Unlike higher plants, seaweeds, most of which display alternation of generation, have far more complicated life histories due to the flexible relationship among morphological phases, cytological events, and genetic behaviors than generally realized [6]. Many red algae have three generations, two sporophyte (diploid) generations, the carposporophyte and tetrasporophyte, in addition to a gametophyte (haploid) [6], most of which are morphologically macroscopic (e.g., adult thallus in *Porphyra*). The gametophyte and tetrasporophyte phases are usually morphologically similar [7], although they have markedly different physiological behaviors [8]. In some species, the carposporophyte is absent in sexual life history and the male and female gametophytes are vegetatively dimorphic [9] (Figure 1A). The life histories in brown algae may include heteromorphic (*Saccorhiza polyschides*, *Laminaria*), monophasic (*Compsonema saxicola*), and isomorphic types (*Ectocarpus siliculosus*), showing great variability between different groups [10]. In genus *Laminaria*, it consists of a macroscopic diploid generation, producing sporangium in which the cells then divide into haploid zoospores by meiosis prior to being released and growing to microscopic dissimilar male and female gametophytes [11] (Figure 1C). The sexual reproduction in the life history of green algae can be isogamous, anisogamous, or oogamous [12]. *Ulva* undertakes an alternation between macroscopic isomorphic diploid and haploid phases, however, in some species there can be twelve morphologically identical phases that differ in cytological and reproductive details [13] (Figure 1B). One of the most fundamental features of seaweeds is their great variety of life histories, running the entire gamut from haploid dominance to diploid dominance [14]. 

The traditional seaweed industry includes alginate production from brown algae and agar and carrageenan production from red algae. Approximately 1 million tons of seaweed can produce approximately 55,000 tons of these hydrocolloids with a value of US $ 585 million [17]. Currently, with the rise of world energy demands and depletion of fossil fuel resources, seaweed is receiving increasing attention as an attractive renewable feedstock for producing fuels and chemicals [18,19,20]. The global farming production of seaweed in 2010 was 19 million tons, with a total value estimated at US $5.7 billion, which is an increase of 30 percent from 2008 [21]. Countries in East and Southeast Asia are leaders in seaweed farming and European countries are using seaweed as a raw natural resource [22]. However, the global scale of seaweed cultivation is still small compared with its increased demand as an alternative renewable fuel [19]. 

The rapid development of seaweed genetic engineering and the establishment of seaweed expression systems are needed because of the agricultural demands on breeding, the production demands of the industrial and biomedical fields, and the environmental demands for bioremediation [23,24,25,26]. Additionally, the use of seaweed genetic engineering could be used as a genome editing method to help scientists decipher the connection between genotype and phenotype, and as a *de novo* genome design tool in synthetic biology from modules to systems [27]. Nevertheless, this field is not fully developed and there currently is not an established seaweed gene expression system, although several transformation methods in seaweeds have been developed [23,28]. The primary purpose of this paper is not to review recent literature in the field of seaweed genetic engineering [23,26,27,28,29], but to highlight gaps in knowledge; especially the rapid development of genome engineering and seaweed genomic research, the necessity of new promoter sequence identification and new transformation methods, as well as, engineering design and biosafety assessments for seaweed expression system establishment. 

## 2. Seaweed Genomics and Model Organism Selection in Seaweeds

There is plentiful and complete genomic information for microalgae, whereas the genomic knowledge of seaweed is very limited. The genome of *Ectocarpus siliculosus*, which is highly related to the commercial brown seaweed *Laminaria* spp., sheds light on the physiology and evolution of multicellularity in brown algae. In this genome, extended sets of light harvesting and pigment biosynthesis genes and new halide metabolic processes have been discovered [30]. For carbon storage, the central pathways of carbohydrate and protein glycosylation are well conserved, while a complicated laminarin metabolism replaces glycogen and starch metabolism from the secondary endosymbiont [31]. The first complete nuclear genome of red seaweed *Chondrus crispus* is a compact genome despite its large size (105 megabase pairs [Mbp]) and possesses rare large-scale gene duplications. Similar to the *Ectocarpus* genome, the *Chondrus* genome possesses halogen metabolism mechanisms for adaptation to the tidal coastal environment. The carbohydrate metabolism of *Chondrus* suggests the polyphyly of cellulose synthesis and the mannosylglycerate synthase in red algae potentially originates from a marine bacterium. In evolutionary history, red algae genomes have undergone loss and expansion including; the loss of genes, introns, and intergenic DNA by ecological forces, followed by an expansion of genome size resulting from the activities of transposable elements [32]. The genome of *Pyropia yezoensis*, one of the most commercialized and well-cultivated seaweeds, has also been sequenced. In its 43 Mbp genome, 35% of the genes are functionally uncharacterized, and a second homolog of the phycobilisome-degradation gene, which had been assumed to be chloroplast derived, was found in the nuclear genome. This newly discovered gene may be involved either in phycobilisome photobleaching or in *P. yezoensis* nitrate metabolism [33]. With the significant development in Next-Generation Sequencing (NGS) technologies, the cost, efficiency has decreased and volume of genome sequencing has increased [34,35]. Recently, it has been reported that the whole genome sequencing of the commercially cultivated seaweed, *Saccharina japonica*, whose genome size is 580–720 Mbp [36], has been completed and the total number of genes is estimated to be up to 35,725; larger than any other eukaryotic algae [37]. In addition to providing basic genomic information for seaweeds that have genomes that are usually large [30,32] and have noise from symbiotic bacteria [33], the decreased cost and increased sequencing efficiency and quality of NGS make it possible to examine species or different strains besides the typical model organisms; providing a new opportunity for comparative genomics within the same phylogenetic seaweed group. Transcriptomic analysis in *Macrocystis pyrifera* assessed gene expression under different abiotic factors such as, light, temperature and nutrients and revealed novel gene families in brown algae. The assembly of the 228 Mbp sequence revealed high genetic similarity between *Macrocystis pyrifera* and its brown seaweed relatives *Ectocarpus* and *Laminaria* [38]. These breakthroughs may produce complete seaweed genomes that could shed light on physiology, ecology, reproduction, evolution, *etc.*, which are essential in genetic engineering.

One bottleneck in seaweed genetic engineering is the diversity of genetic backgrounds and physiological activities (life history) among different seaweed strains. Both transient and stable genetic transformations have been accomplished in only a few strains of red, green, and brown seaweeds [23,28]. At present, only a few genii of seaweeds (well-commercialized ones or ones with long research histories) have been cultivated in the laboratory. Some materials for seaweed genetic engineering are directly field-isolated. A small change in genome or life history could lead to alterations in the parameters of genetic engineering experiments. To some extent, this prevents the spread of established genetic transformation methods to other seaweeds. It also hampers research into the fundamental mechanisms and principles of seaweed genetic transformation; thus some techniques remain at the technological level. With the successful assembly and analysis of the complete genomes stated in last paragraph, it is possible to establish several model organisms in seaweeds. *Ectocarpus* is regarded as the first promising candidate for consideration since its 214 Mbp genome has been assembled and analyzed [30] and several other genetic datasets or methodologies are available [39,40]. The *Ectocarpus* genome is much smaller than its brown relatives Laminariales (580–720 Mbp) [34] and Fucales (1095–1271 Mbp) [41]; thus it should be more amenable to genome screening and manipulation. In addition, there are ~1800 well-maintained *Ectocarpus* strains in several collection centers and a barcode system for those strains is under construction [42]. In the future, we can expect the ability to conduct comparative genomic studies among these genetic resources, due to the decrease in cost and increase in efficiency brought by NGS. A set of laboratory protocols ranging from high-quality DNA extraction to seaweed cultivation [42,43,44,45,46,47] for *Ectocarpus* have also recently been published. In red seaweed, *Pyropia yezoensis* and other *Porphyra* spp. have been proposed as model organisms in red seaweed research of life history and ecophysiology [33,48].

## 3. Genome Engineering

Genome engineering is focused on the development of methods to precisely manipulate nucleic acids in living cells. Targeted genome modification in plants has been regarded as an elusive goal [49]. However, recent advances in sequence-specific genome engineering technologies have enabled the control of genetic material via targeted genome modifications [50] in model plant organisms. These tools can be grouped into two categories: protein-directed and nucleotide-directed specificities [51]. zinc finger nucleases (ZFN) have been successfully applied in *Chlamydomonas* [52], however, ZFN technology suffers from difficulties in design, construction, cost and uncertain success rates [53,54]. Here we are going to focus on two newly developed targeted genome modification tools. The first is protein directed transcription activator-like effectors (TALEs or TALs), the second is RNA-directed type II prokaryotic clustered regularly interspaced short palindromic repeats (CRISPR). For other target genomic engineering tools such as ZFN, readers may refer to several excellent and comprehensive reviews summarizing recent advances in precise genome editing technology [50,51,55,56]. 

**Figure 2:**
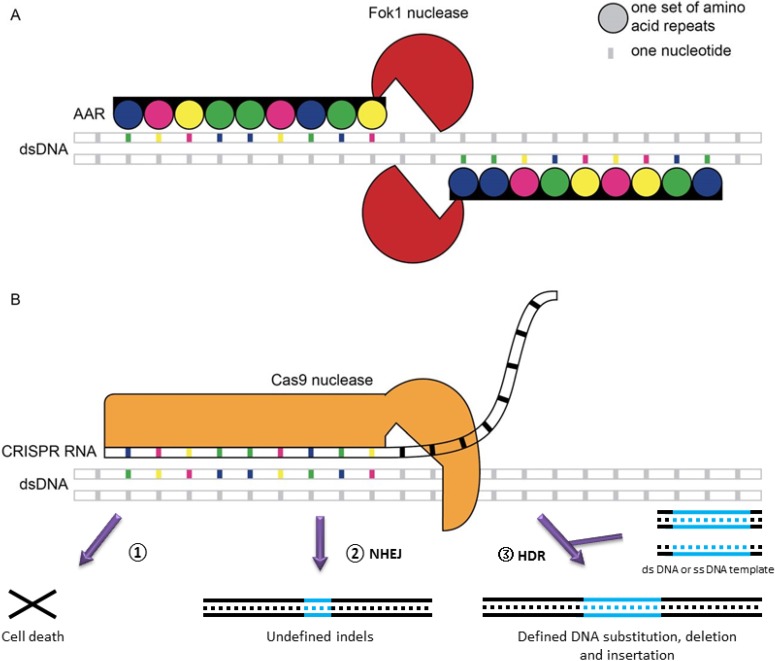
Schematic diagram comparing TALE and CRISPR genome editing technologies, adapted from [86] with the permission from Journal of Experimental Botany. (**A**) TALE. (**B**) CRISPR, the genome breaks led by CRISPER will generate three possibilities: (1) cell death when dsDNA break are not moved, (2) undefinded indels by NHEJ, (3) homology-directed repair [70].

TALs [57] have rapidly developed and have been utilized to create site-specific gene-editing [58] (Figure 2A). TALs are proteins produced by the pathogenic plant bacteria, *Xanthomonas*, when they infect plants through the type III secretion pathway [59]. These proteins can activate the expression of plant genes by binding effector-specific DNA sequences through their central tandem repeat region where the 12th and 13th amino acid diresidue corresponds to a specific nucleotide sequence [60] and transcriptionally activates gene expression. The TAL technique is more cost effective, has simpler design requirements and lower off-target activities than ZFN [61]. Due to the convenience of engineering new DNA binding specificities compared with ZFN [62], it has been applied in model plants to alter reporter-gene expression in tobacco [54,63], insertions and deletions in *Arabidopsis thaliana*, gene knockouts in rice [64], and the generation of disease-resistant rice [58]. There also have been calls for this technology to be applied to microalgae homologous recombination [65]. Several in-house methods of synthesizing TAL proteins to target specific DNA sequences in *Chlamydomonas* have been established, fused with *Fok*I nuclease, this technology could be utilized to create specific modifications in the *Chlamydomonas* genome [66,67]. 

CRISPR has been hailed as a revolution in genomic engineering [68] (Figure 2B). It is a bacterial and archaeal immune system that exists in 40% of eubacteria and 90% of archaea [69] and consists of three core components: RNA guided CRISPR associate protein (Cas9) nuclease, CRISPR RNA (crRNA) and trans-acting crRNA (tracrRNA) [70], although occasionally the latter two components can be fused into a single guide RNA (sgRNA) [71]. CRISPR can directly edit the genome by either Non-Homologous Ending Joining (NHEJ), producing undefined indels, or template-dependent Homology-Directed Repair (HDR), which leads to a defined DNA substitution, deletion and insertion [70]. In *Arabidopsis*, the efficiency of HDR-mediated insertion is higher than NHEJ-mediated insertions [72]. When CRISPR fuses with effectors or transcriptional repressor domains, it generates stable and efficient transcriptional repression or activation, or robustly silences multiple endogenous gene expression in human and yeast cells [73]. This system has been successfully used in plant organisms to perform gene modification and mutagenesis [74,75], multiplex gene editing [72,76], genome-scale screening [77,78], transcriptional control (up-regulated and down-regulated) [73], dynamic imaging of chromosome activity [79], and even multiplexed RNA-guided transcriptional activation, repression and gene editing simultaneously [80]. Presently, the majority of studies using CRISPR engineering that have been related to genome screening and transcriptional regulation have been performed in bacterial or animal cells [51,70].

Realistically, both the TAL and CRISPR techniques are not mature enough to be applied universally. Efforts must be made to resolve many fundamental problems in both methods; for example, the molecular structure and catalytic mechanism of CRISPR complex [70] and the pathway of TALs delivery into cells by lentiviruses [55]. Additionally, studies need to be performed for these targeted genome editing tools to determine their safety and specificity to decrease off-target possibilities [81,82] and to compare their efficiencies [55]. However, it is not too soon to begin genome editing studies in seaweed, although the aforementioned pioneering works in algae have primarily been performed in the model microalga *Chlamydomonas*. Based on the assumption that closely phylogenetically related species may share similar genetic, biochemical and physiological and morphological features [83]; the establishment of genome editing systems may derive from research in unicellular microalgae such as, *Porphyridium purpureum* [84] in Rhodophyta and *Thalassiosira pseudonana* [85] in Heterokontophyta. 

## 4. Natural Promoter Identification and Promoter Engineering

Eukaryotic promoters are generally described as having a core promoter near the site of transcription initiation and one or more enhancer elements that may be located more distantly [87]. Promoters have guided evolution for millions of years as the primary mechanism responsible for the integration of different mutations favorable for the environmental conditions [88]. Because promoters are in non-coding regions, they are subject to less stringent evolutionary selection than protein-coding regions and have greater probability of nucleotide substitution [89]. The best characterized core promoter element is the TATA box, discovered in 1979 in *Drosophila* [90], which also exists in most promoters used in seaweed genetic engineering [28]. Nevertheless, TATA boxes are not present in all promoter regions—only approximately 10%–20% of metazoan core promoters [91] and only 29% *Arabidopsis* promoters have TATA boxes [92]. It seems that the organization of core promoters is phylogenetically different among different organisms [88], and sometimes significantly different between animals and plants [92]. When compared with higher model plants and animals, it is obvious that many core promoter elements remain undiscovered in marine algae.

The traditional viral promoters CaMV35S and SV40, commonly used in plant and animal genetic engineering, have been broadly applied in seaweed genetic engineering when there is sparse genetic information available [74,93,94,95]. These two promoters are typical eukaryotic class II promoters with a TATA box. However, exogenous proteins are often difficult to express when they do not originate from the target organism. In unicellular green algae models, some heterologous promoters allow transient gene expression, but the efficiency of the reporter and marker genes is low [96,97]. The same low efficiency of expression is observed in red seaweed. A considerably low activity of the CaMV35S promoter is observed in *P. yezoensis* cells [98]. Some algal endogenous promoters, such as the diatom-originated fucoxanthin-chlorophyll a/c binding protein gene (*fcp*) and the endogenous 5′ upstream region of the actin 1 gene from *P. yezoensis* (PyAct1), have shown efficient activity when used to construct expression vectors. The former has been applied in *L. japonica* [99], whereas the latter has been found effective in 12 red seaweed species [100,101]. The successful application of the PyAct1 promoter indicates that extensive study should be devoted to the search for and design of endogenous promoters within seaweed genomes. This necessity arises because exogenous promoters usually have different structures of DNA sequence and apply different transcriptional regulation of protein-coding genes. Promoter trapping is one of the methods used for novel promoter isolation and characterization in plants [102], and it has been successfully applied in modeling the green alga, *Chlamydomonas reinhardtii* [103]. Increasing numbers of assembled marine algal genome sequences are now available due to the high sequencing throughput brought about by NGS technologies. Presently, there are approximately 19 marine eukaryotic algae genomes that are finished being sequenced or are in the process of being sequenced [33,37,84,104]. Natural promoter sequence identifications have not been comprehensively conducted in marine algae, but with the help of NGS and bioinformatic technologies the situation is improving. The completion of the algal genomic assemblies will provide a foundation for the comparative analysis of gene regulation networks to determine the *cis*-regulatory code [105], which will allow the discovery of additional endogenous promoters and viral or phage-derived heterologous promoters. 

Natural promoters for use in seaweed genetic engineering can either be isolated from endogenous algal sequences or isolated from a heterologous viral promoter. Occasionally, heterologous viral or phage-derived promoters cannot regulate transcription levels, meanwhile, isolated endogenous promoters are commonly unable to maximize the transcriptional capacity of the host [106]. These shortcomings do not allow for fine-tuned control of transcription, which prevents their use in complex metabolic and genomic engineering applications. Promoter engineering may overcome these difficulties and possibly will allow the optimization of metabolic pathways in cooperation with other methods including; synthetic ribosome-binding sites design [107], mRNA stability improvement [108], RNase III activity modulation [109], codon usage [110], *etc.* At present, there are four prevailing strategies in rational promoter design; introducing random mutations into the promoter sequence by error-prone PCR, mutating non-consensus spacer nucleotide regions within a promoter via saturation mutagenesis, assembling different tandem upstream activation sequences to tune the core promoter and altering the structure of transcription factor-binding site sequences [106]. Schlabach and colleagues have established a library of possible 10-mer DNA sequences in tandem from the upstream activation sequence of one promoter to generate stronger enhancement activity for this promoter using a synthetic biology approach [87]. All of these methods make it possible for *de novo* tunable promoter synthesis in seaweed genetic engineering, while more rational strategies will still rely on the increasing understanding of promoter sequence structure diversity from marine algae uncovered by NGS technologies. 

Codon usage is another barrier to the transport of exogenous protein-coding genes into seaweeds. Exogenous proteins usually contain codons which are rarely used in the desired host, in addition to transcriptional regulation elements within their own coding sequence [111]. Codon bias is sometimes regarded as the single most important determinant of exogenous protein expression [112,113]. Optimizing the codon usage of algae-destined transgenes may increase the expression efficiency of proteins and decrease their susceptibility to gene silencing [114]; such optimizations may be necessary for high-level protein expression for commercial purposes [115]. The vectors containing artificially optimized GUS coding sequences with CaMV35S, GAPDH, or PyAct1 promoters show higher expression efficiencies than those of each promoter and the original GUS gene [98]. A cyan fluorescent protein modified according to the codon usage of *P. tenera* and *P. yezoensis* has been successfully used in transgenic research; this fluorescent protein uses short emission wavelengths to diminish the background fluorescence interference from chlorophyll molecules in algal cells [116]. Aside from its original use in gene amplification, *de novo* gene synthesis has become another powerful tool to express heterologous genes in host organisms. Designing an optimal gene requires a comprehensive inspection of codon usage, mRNA secondary structure, cryptic ribosome binding sites [117], and the interaction of the target gene with the cellular environment of the host. A rational way to optimize the criteria and algorithms for heterologous gene expression has recently been proposed [118]. These optimized algorithms are essential in the development of synthetic biology for use in seaweed biotechnology [20].

## 5. Transformation Methods

**Figure 3:**
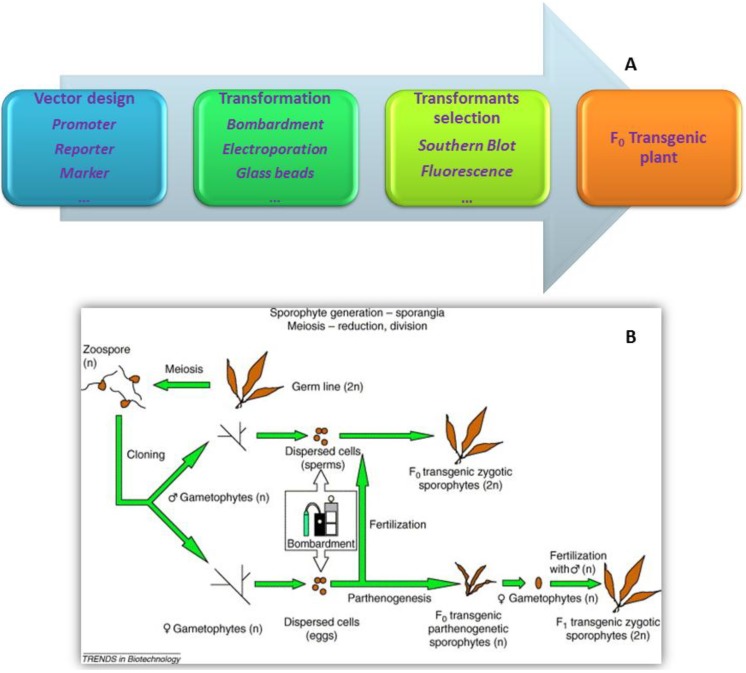
Workflow of seaweed genetic engineering. (**A**) General workflow; (**B**) Workflow in brown seaweed *Laminaria japonica* (adapted from [24] with permission of Trends in Biotechnology).

Particle bombardment, electroporation, and glass beads are the three primary transformation methods applied in seaweed due to their mature technologies and their successful application in many phylums and classes of seaweeds (red, green, and brown seaweeds) [28,74,100,119,120,121] (Figure 3). Details on these methods are available in the literature [23]. In some higher plants, the *Agrobacterium*-mediated method yields high proportions of transformants that combine low copy number with the expression of the non-selected reporter gene, and bombardment particles with minimal cassettes yield high absolute transformation efficiencies [122]. However, the scarcity of studies on the *Agrobacterium*-mediated method [123] in seaweed and the limited understanding of the *Agrobacterium* mechanism within the algal nuclear genome constrain the application of the Agrobacterium-mediated method in seaweed [23].

Large dsDNA viruses known to infect eukaryotic algae are categorized into two groups: viruses in the unicellular microalgae *Chlorella* system and viruses in the brown seaweed system [22]. Nearly twenty years ago, virus-mediated transformation was successfully applied in filamentous brown seaweed [124]. However, there are few follow-up studies on this transformation method. Viruses that infect brown algae have different sizes, diameters, and host specificities [125]. When considering a rational and quantitative virus-infected seaweed genetic transformation, at least three factors need to be considered in advance. The first factor is the minimum threshold abundance at which seaweed could be infected by a virus. This minimum infection threshold universally exits in nature [126]. The transport of a virus into a small aquatic organism can be described quantitatively as the diffusive transport from solution [127]. Thus, the viral infection process (transport of virus and host-virus interaction) in the gametophyte of seaweeds can be mathematically modeled. Most of the aquatic host-virus interaction dynamic models introduce a parameter of host cell death rate due to lysis [128,129], but the viruses that infect brown algae are usually benign and nonlethal [130]. The second factor is the search for a universal nonlethal virus that targets seaweeds. The brown algae is the only class of eukaryotic algae whose species can be infected by an algal virus; in contrast to other cases in which one virus specifically infects a single host species, more than one species of the brown algae can be infected [130]. The virus in the brown algal system is generally benign, but other algal viruses might lead to algal lysis and cause death, which is not expected in genetic engineering. The third and last factor is the infection mechanism. Studies have confirmed that some brown algal viruses maintain the amplification of their DNA, along with algal cellular mitotic divisions, in unicellular gametes or spores and do not form viral particles until the cells develop into a mature organ (gametangia or sporangia) [131]. Details on the mechanism of viral DNA combination and modification to algal genome may be elucidated with the development of genomic tools from genome to phenome.

Transposons, a group of mobile DNA first discovered in maize, have been developed as powerful tools for genetic engineering in some microalgae [132,133]. The high transgenic efficiency of transposons can, to a certain extent, avoid the silencing of exogenous genes in host cells. The recently completed seaweed genomes discussed in Section 2, especially the genome of *C. crispus*, implies the existence of many transposon elements. The transposon component of this genome is quite complicated because of their huge number and significant divergence [32]. Transposons were also detected and identified in the *Pyropia yezoensis* genome [33]. These potential endogenous elements will “open a new window” because seaweed genomes are essentially expanded or shrunk to some extent via several endosymbiotic events in evolutionary history [2,134] as a partial consequence of transposable elements [32].

## 6. Biosafety Assessments of Transgenic Seaweeds

The initial debate about the risks from transgenic organisms emerged with the birth of the first genetically modified (GM) organism in the 1970s [135]. The threat to biodiversity, gene flow, and resistance risk are the main concerns revolving around transgenic plants, including GM seaweeds. We cannot be less cautious when GM organisms are modified by precise editing, replacement or insertion of genes without the use of any selectable markers and to specifically select the targeted genomic region [136]. Some scientists have predicted that most target GM microalgal traits (here, microalgae are morphologically similar to spores from seaweeds) are hard to maintain in nature and would rapidly diminish; they have also emphasized the need for a rigorous evaluation of such traits at the mesocosms scale to monitor their continuous gene flow from GM algae to the environment [137]. In addition, risk analysis should be straightforward at the species level to evaluate its impact on indigenous species [138] or even at the population level when the wild variety of GM seaweed is taken from the local environment. Lessons on scientific risk evaluation and management could be learned from higher plants [139,140,141]. However, unlike land plants, GM seaweeds cannot avoid escaping to the environment because of constant water flow. This condition leads to gene flow, especially when GM seaweeds become sexually mature and release their spores. 

We propose a set of rational assessment programs for evaluating gene flow from GM seaweeds to local or large-scale environment based on the assessment of gene flow probability from transgenic plants [140]. However, necessary modifications are added considering the different fluid features of aquatic environments compared with terrestrial environments and the complex biological and ecological characteristics of seaweeds. This set of programs includes the following. (1) Biology of target seaweed: Fundamental information of genetic structure (endogenous plasmids, reproduction-related genes, *etc.*), life cycle, and maximum growth conditions will have a primary effect on the probability and degree of the gene flow from GM seaweeds; (2) Distribution medium of other aquatic organisms: The life cycle of seaweeds is so complex that any organism, including marine invertebrates, vertebrates, or even other algae and sea grasses, can assist GM seaweeds in the distribution of GM spores; (3) Hydrological structure of the local aquatic environment: The trial area should be a relatively stable environment; however, monthly or seasonal changes should be considered, and daily or weekly hydrological fluctuations must be noted to understand the time of the release of spores; (4) Genetic information exchanges with other related population or species: Seaweeds can “compare notes” on their respective genetic information by various sexual or asexual reproductive processes according to the degree of their genetic compatibility to other species or populations; (5) Life cycle and reproductive strategies of GM seaweeds: Close attention should be given to any genetic, physiological, or morphological changes in the life history of GM seaweeds, especially during their release of spores to the adjacent aquatic environment; (6) Survivability of algal propagules from GM seaweed: The fitness of any escaped vegetative fragments or sporangia of GM seaweeds to the environment outside the cultivation area must be determined; (7) Semi-quantification of gene flow: Several mathematical models specific to each GM species or population are required because the life cycles of seaweeds are extremely diverse; even within a genus, the life cycle can significantly differ under various conditions [142]. In the present work, we use the term “semi-quantification” instead of “quantification” as mentioned in the study of Chandler [140] because this process is more complex within aquatic systems than within land systems [143,144]; (8) Post-release mechanism of gene dispersal from GM seaweed: Although this program would only be effective after obtaining permission to cultivate GM seaweed in the open sea, it can never be overemphasized because local cultivation may lead to geographical gene flows along the hydrological structure of the sea or the GM seaweed could randomly be water ballasted to other seas similar to the mechanism of other aquatic invasive organisms.

Another necessary step is to construct “complete marine algae-derived” vectors, *i.e.*, endogenous promoters, reverse mutation as a selective marker, and the replacement of any non-algal viral DNA sequence with algal virus nucleotides [23]. Basing on the extensively studied theory of “paradox of the plankton” put forward by Hutchinson [145], Gressel emphasized the “gene mitigation” strategy originating from transgenic higher plants and introduced several mutation strategies to cause GM algae to be unfit to reproduce in nature and become incapable of adapting to local ecosystems [146].

## 7. Conclusions

Seaweed genetic engineering could bridge the gap between fundamental and applied studies in seaweed research. The decreasing cost of sequencing provides us with many opportunities to investigate the fine genetic structure of seaweeds and consequently identify innovative genetic transformation elements (promoters, transformation methods, selective markers, *etc.*). The final biosafety assessment of a GM seaweed demands multi-disciplinary research on algal genetics, physiology, reproductive biology, and ecology from the molecular to at least the local aquatic ecosystem level through mathematics, biology, chemistry, physics, and even sociology when considering environmental issues. These innovations will bring unlimited possibilities in seaweed genetic engineering, which is still in its infancy. Combining these innovations with the rapidly developing fields of systems biology and metabolic engineering will satisfy the demands related to energy, environment, and human health.
